# Holmium Laser Enucleation versus Transurethral Resection in Patients with Benign Prostate Hyperplasia: An Updated Systematic Review with Meta-Analysis and Trial Sequential Analysis

**DOI:** 10.1371/journal.pone.0101615

**Published:** 2014-07-08

**Authors:** Sheng Li, Xian-Tao Zeng, Xiao-Lan Ruan, Hong Weng, Tong-Zu Liu, Xiao Wang, Chao Zhang, Zhe Meng, Xing-Huan Wang

**Affiliations:** 1 Department of Urology, Zhongnan Hospital, Wuhan University, Wuhan, People's Republic of China; 2 Center for Evidence-based Medicine and Clinical Research, Taihe Hospital, Hubei University of Medicine, Shiyan, People's Republic of China; 3 Department and Institute of Hematology, Union Hospital, Tongji Medical College, Huazhong University of Science and Technology, Wuhan, People's Republic of China; University of British Columbia, Canada

## Abstract

**Background:**

Holmium laser enucleation (HoLEP) in surgical treatment of benign prostate hyperplasia (BPH) potentially offers advantages over transurethral resection of the prostate (TURP).

**Methods:**

Published randomized controlled trials (RCTs) were identified from PubMed, EMBASE, Science Citation Index, and the Cochrane Library up to October 10, 2013 (updated on February 5, 2014). After methodological quality assessment and data extraction, meta-analysis was performed using STATA 12.0 and Trial Sequential Analysis (TSA) 0.9 software.

**Results:**

Fifteen studies including 8 RCTs involving 855 patients met the criteria. The results of meta-analysis showed that: a) efficacy indicators: there was no significant difference in quality of life between the two groups (P>0.05), but compared with the TURP group, Qmax was better at 3 months and 12 months, PVR was less at 6, 12 months, and IPSS was lower at 12 months in the HoLEP, b) safety indicators: compared with the TURP, HoLEP had less blood transfusion (RR 0.17, 95% CI 0.06 to 0.47), but there was no significant difference in early and late postoperative complications (P>0.05), and c) perioperative indicators: HoLEP was associated with longer operation time (WMD 14.19 min, 95% CI 6.30 to 22.08 min), shorter catheterization time (WMD −19.97 h, 95% CI −24.24 to −15.70 h) and hospital stay (WMD −25.25 h, 95% CI −29.81 to −20.68 h).

**Conclusions:**

In conventional meta-analyses, there is no clinically relevant difference in early and late postoperative complications between the two techniques, but HoLEP is preferable due to advantage in the curative effect, less blood transfusion rate, shorter catheterization duration time and hospital stay. However, trial sequential analysis does not allow us to draw any solid conclusion in overall clinical benefit comparison between the two approaches. Further large, well-designed, multicentre/international RCTs with long-term data and the comparison between the two approaches remain open.

## Introduction

The latest American Urological Association's (AUA) Guideline defines transurethral resection of the prostate (TURP) as the “gold standard” surgical treatment for benign prostate hyperplasia (BPH) [1]. However, the latest guideline from the European Association Urology (EAU) indicates that when the prostate volume is larger than 80 ml, it is dangerous for BPH patients to be treated with TURP, and EAU recommends holmium laser enucleation of the prostate (HoLEP) [2]. Holmium laser techniques have been introduced as a surgical intervention for BPH more than 15 years. In 1997, Gilling et al [3] conducted the first prospective randomized controlled trial (RCT) comparing TURP with holmium laser resection of the prostate (HoLRP), the result revealed HoLRP was associated with significantly longer mean resection time (42.1 vs. 25.8 minutes) when compared to TURP, while symptomatic and urodynamic improvement were equivalent in both groups. Subsequently, HoLRP combined with transurethral tissue morcellation evolved into HoLEP. Since then, many studies on this issue have been conducted with different or even contradictory results [4]–[6]. Therefore, whether HoLEP is non-inferiority, equivalence, or superiority to TURP remains unclear. An in depth reassessment of this question has important clinical implications. Consequently, we performed this systematic review with meta-analysis and trial sequential analysis (TSA) of all the published RCTs in the hope of providing more precise evidence.

## Methods

We reported this systematic review and meta-analysis based on the methodology recommended by the Cochrane Collaboration and according to the Preferred Reporting items for Systematic Review and Meta-analysis (PRISMA) statement [7]. The protocol (CRD42014007334) of this systematic review was published in the PROSPERO register (www.crd.york.ac.uk/PROSPERO).

### Eligibility criteria

Studies were eligible for inclusion if they met the following criteria: (1) study participants were clearly diagnosed as BPH and needed surgical treatment (we excluded patients who had unstable bladder, neurogenic bladder, preoperative urethral stricture, history of bladder cancer, or previous history of bladder neck cancer surgery); (2) randomized controlled studies which used HoLEP and TURP as the intervention and control arms, respectively; (3) at least reported one of the efficacy, safety or perioperative outcomes, which consisted of the International Prostate Symptom Score (IPSS), maximum flow rate (Qmax) (ml/s), quality of life (QoL), postvoid residual volume (PVR) (ml), the International Index of Erectile Function (IIEF), blood transfusion, TUR syndrome, urethral stricture, bladder neck contracture, secondary treatment, acute urinary retention (AUR), urinary tract infection (UTI), and transient hematuria, operating time (min), catheterization time (h), hospital stay (h), reduction of haemoglobin (g/dl) and serum sodium (mmol/L).

### Search strategy

We searched PubMed, EMBASE, Science Citation Index, and the Cochrane Library for relevant published studies up to October 10, 2013 (updated on February 5, 2014). The search strategy was summarized in Appendix S1. The bibliographies of the included studies and recent reviews were hand-searched. No language restriction was applied.

### Study selection and data extraction

Our systematic search approach yielded titles and abstracts of published articles according to the above eligibility criteria and we excluded the clearly irrelevant results. The remaining trails were evaluated in full text. Information of each included trial was extracted using a pre-made data extraction form. We extracted the following trial characteristics: first author's name, publication year, country, and the detailed information of PICOS (participant, intervention, comparison, outcomes, and study design). For any missing data, we contacted the corresponding authors. Two authors independently selected study and extracted data, any disagreement was resolved by discussion.

### Methodological quality assessment

The methodological quality of included studies was evaluated using the Cochrane collaboration's tool for assessing risk of bias [8]. We mainly assessed the following six items: adequate sequence generation, allocation concealment, blinding, incomplete outcome data addressed, reporting bias, and other bias. Each item was answered by “Low” (low risk of bias), “Unclear” (either lack of information or uncertainty over the potential for bias), and “High” (high risk of bias).

### Statistical analysis

All data were pooled using STATA version 12.0 (Stata Corp). For binary outcomes, relative risks (RRs) and corresponding 95% confidence intervals (CIs) were calculated; for continuous outcomes, weighted mean differences (WMDs) and their 95% CIs were calculated. The Cochran Q test was used to explore statistical heterogeneity with P<0.1 for statistical significance; a quantitative measure of heterogeneity across studies was also investigated using the I^2^ statistic. Studies with I^2^ values of less than 40% were considered as having acceptable level of statistical heterogeneity [9]. We used a fixed-effect analytical model to pool the results of studies with acceptable or no heterogeneity. Subgroup analysis was conducted to investigate potential source of between-study heterogeneity. A two-side P value <0.05 in the Z-test was regarded as statistically significant.

### Trial sequential analysis

Cumulative meta-analyses of trials are at risk of yielding random errors because of sparse data and repetitive testing of accumulated data [10]–[16]. In the single trial, trial sequential analysis (TSA) is similar to interim analysis that may increase the risk of type I errors. In order to minimize this risk, monitoring boundaries were applied to determine if the trial should be terminated early under the condition of an amply small P value [17]. In the same way, trial sequential analysis can be applied to meta-analysis [10], [14]–[15], [18]. Trial sequential analysis depends on the quantification of the required information size. We calculated the required information size adjusted for diversity since the heterogeneity adjustment with I^2^ underestimate the required information size [16]. The trial sequential analysis was performed to maintain an overall 5% risk of a type I error and 20% of the type II error (a power of 80%) [16]. We anticipated an intervention effect of a 20% relative risk increase for the calculation of the required information size [13]. We conducted post hoc trial sequential analysis with 35% relative risk increase if the required information size was very large. For the continuous outcomes of IPSS, Qmax, PVR, duration of operation, catheterization time, hospital stay, and reduction of haemoglobin, we estimated the required information size to reject a reduction of 0.5, 3.0 ml/s, 5.0 ml, 5.0 min, 5 h, 5 h, 0.5 g/dl, respectively. We applied a constant continuity correction of 1.0 in the no event trial. We used software Trial Sequential Analysis (version 0.9, http://www.ctu.dk/tsa/) and provided the 95% confidence intervals adjusted for sparse data or repetitive testing.

## Results

### Characteristics of included studies

**Figure 1:**
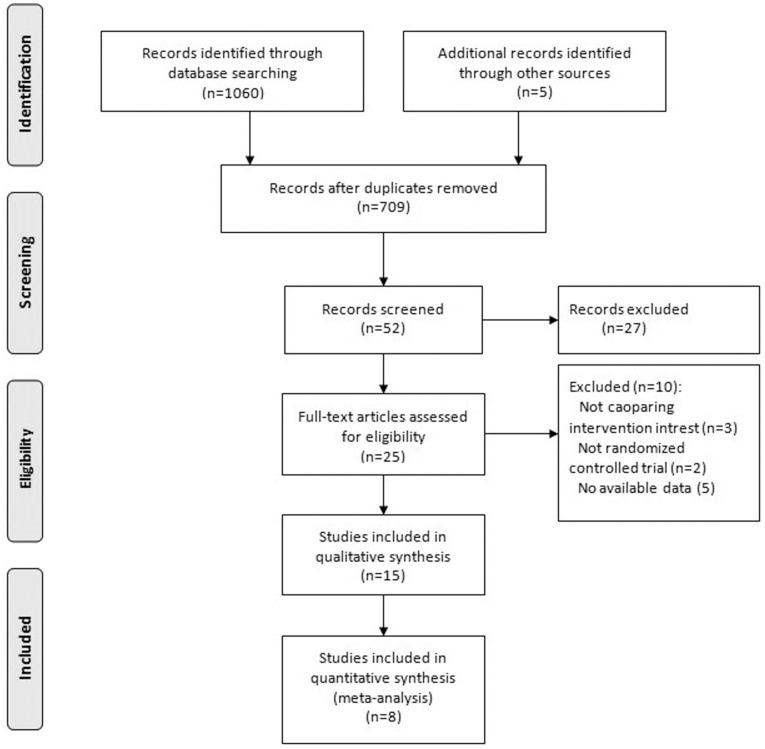
Identification of eligible studies.

Our initial search yielded 1065 potential publications and finally 8 trials [19]–[26] were included (Fig. 1). The eight trials [19]–[26], which were referring to fifteen publications [19]–[33] based on the different durations of follow-up period. Our meta-analysis included data of 855 participants. All trials were published in English. Table 1 shows the baseline characteristics of the included RCTs. The max follow-up duration ranged from 9 months to 24 months.

**Table 1:** Characteristics of the included randomized controlled trials (RCTs).

Trial	Publication, yr	Sample size	Follow-up, mo	Age, yr	Prostate size, g	IPSS	QoL	Qmax, ml/s	PVR, ml	IIEF
Gilling/Fraundorfer/Westenberg et al. [21], [28], [31]	1999; 2001; 2004	61/59	12	66.9/66.8	44.3/44.6	21.9/23	NA	8.9/9.1	87.8/84.7	NA
Tan/Gilling/Wilson et al. [26], [29], [32]	2003; 2006; 2012	31/30	12	71.7/70.3	77.8/70	26.4/23.7	4.8/4.7	8.4/8.3	116.1/126.7	NA
Kuntz/Ahyai et al. [23], [27]	2004; 2007	100/100	12	68.0/68.7	53.5/49.9	22.1/21.4	NA	4.9/5.9	238/216	NA
Montorsi/Rigatti/Briganti et al. [25], [30], [33]	2004; 2006; 2006	52/48	24	65.1/64.5	70.3/56.2	NA	NA	NA	NA	22.3/21.4
Gupta et al. [22]	2006	50/50	12	65.9/65.7	57.9/59.8	23.4/23.3	NA	5.2/4.5	112/84	NA
Mavuduru et al. [24]	2009	15/15	9	69.9/66.5	36.5/36.3	22.5/21.4	NA	5.8/6.9	91/103	NA
Eltabey et al. [20]	2010	40/40	12	67.5/68.3	62.4/58.5	23/25	NA	8.4/8.1	130/105	NA
Sun et al. [19]	2014	82/82	12	72.2/71.9	NA	24.4/24.6	4.6/4.6	5.3/5.7	115.8/108	NA

IPSS = International Prostate Symptom Score; QoL = quality of life; Qmax = maximum flow rate; PVR = postvoid residual volume; IIEF = International Index of Erectile Function; NA = not available.

### Bias risk assessment

The risk of bias could be fully assessed in only one trial [26] and it was judged to be of low risk of bias in all items. Two trials [22], [25] did not report the method of randomization. Method of blinding was given in two trials, of which one [19] blinded the study participants and outcome assessors and another [26] blind the outcome assessors only. Table 2 illustrates the risk of bias assessment results.

**Table 2:** Risk of bias assessment of the included randomized controlled trials (RCTs).

Trial	Random sequence generation	Allocation concealment	Blinding	Incomplete outcome data	Selective outcome reporting	Baseline imbalance	Other bias
Gilling/Fraundorfer/Westenberg et al. [21], [28], [31]	Low	Unclear	Unclear	Low	Low	Low	Low
Tan/Gilling/Wilson et al. [26], [29], [32]	Low	Low	Low	Low	Low	Low	Low
Kuntz/Ahyai et al. [23], [27]	Low	Unclear	Unclear	Low	Low	Low	Low
Montorsi/Rigatti/Briganti et al. [25], [30], [33]	Unclear	Unclear	Unclear	Low	Low	Low	Low
Gupta et al. [22]	Unclear	Unclear	Unclear	Low	Low	Low	Low
Mavuduru et al. [24]	Low	Unclear	Unclear	Low	Low	Low	Low
Eltabey et al. [20]	Low	Unclear	Unclear	Low	Low	Low	Low
Sun et al. [19]	Low	Unclear	Low	Low	Low	Low	Low

### Efficacy

#### IPSS

**Figure 2:**
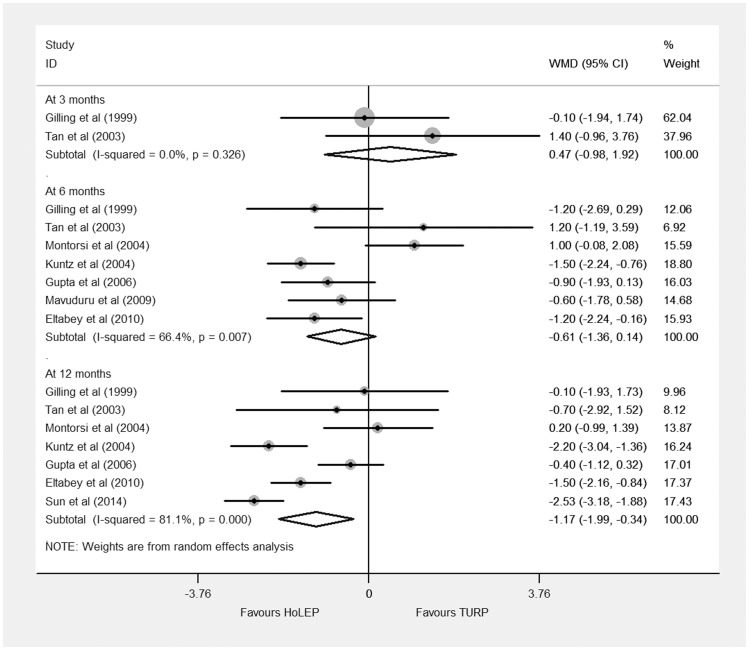
Forest plot for International Prostate Symptom Score (IPSS) at 3 months, 6 months, and 12 months based on a random effects model. WMD = weight mean difference; CI = confidence interval.

The IPSS data were acquired from seven trials [19]–[26]. Of them, two [21], [26] reported IPSS at 3 months, seven [20]–[26] at 6 months, and seven [19]–[23], [25]–[26] reported data at 12 months. Meta-analysis of 3-month and 6-month IPSS showed no significant differences (3 months: WMD 0.47, 95% CI, −0.98 to 1.92, heterogeneity I^2^ = 0.0%, TSA adjusted 95% CI, −5.46 to 6.40; 6 months: WMD −0.61, 95% CI, −1.36 to 0.14, heterogeneity I^2^ = 66.4%, TSA adjusted 95% CI, −3.6 to 2.46) (Fig. 2). However, at 12 months, treatment of HoLEP led to a significant decrease in IPSS based on a random effects model (WMD −1.17, 95% CI, −1.99 to −0.34, heterogeneity I^2^ = 81.1%, TSA adjusted 95% CI −4.54 to 2.21) (Fig. 2). Trial sequential analysis of trials data obtained at 12 months showed that there was insufficient evidence to show a reduction of 0.5 in IPSS, the cumulative Z-curve surpassed the futility boundary, but it did not cross the trial sequential monitoring boundary (Fig. S1).

#### Qmax

**Figure 3:**
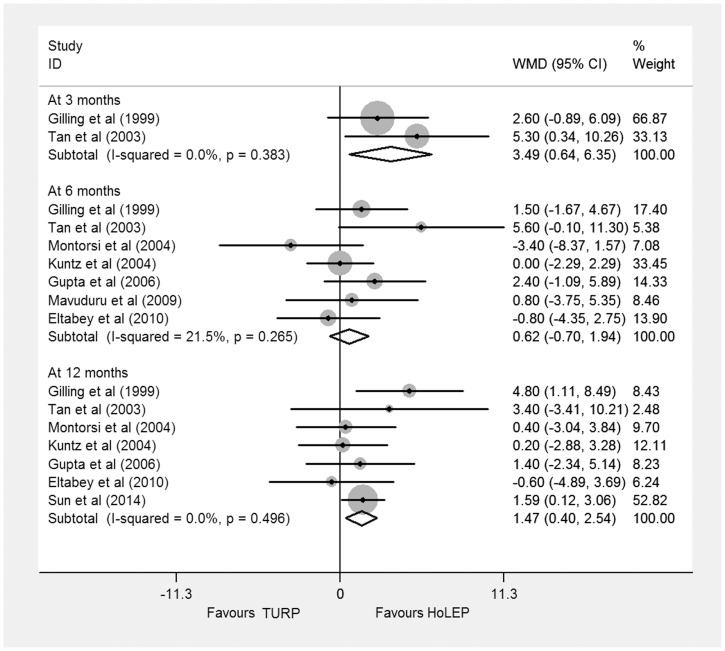
Forest plot for maximum flow rate (Qmax) at 3 months, 6 months, and 12 months based on a fixed effects model. WMD = weight mean difference; CI = confidence interval.

The Qmax data including 855 BPH patients were acquired from eight trials [19]–[26]. Of them, two [21], [26] reported Qmax at 3 months, seven [20]–[26] at 6 months, and seven [19]–[23], [25]–[26] reported data at 12 months. There was no significant difference in Qmax at 6 months (WMD 0.62 ml/s, 95% CI −0.70 to 1.94 ml/s, heterogeneity I^2^ = 21.5%, TSA adjusted 95% CI, −0.62 to 2.02 ml/s). But the results showed significant differences favoring HoLEP at 3 and 12 months based on a random effects model (3 months: WMD 3.49 ml/s, 95% CI, 0.64 to 6.35 ml/s, heterogeneity I^2^ = 0.0%, TSA adjusted 95% CI, −2.45 to 9.64 ml/s; 12 months: WMD 1.47 ml/s, 95% CI, 0.40 to 2.54 ml/s, heterogeneity I^2^ = 0.0%, TSA adjusted 95% CI, −0.75 to 3.91 ml/s) (Fig. 3). Trial sequential analysis of trials data obtained at 12 months showed that there was insufficient evidence to show a reduction of 3.0 ml/s in Qmax, the cumulative Z-curve surpassed the futility boundary, but it did not cross the trial sequential monitoring boundary (Fig. S2).

#### QoL

The QoL data were obtained from four trials including 445 BPH patients. Two trials [21], [26] reported QoL at 3 months, three [21], [25]–[26] at 6 months, and four [19], [21], [25]–[26] at 12 months. Meta-analysis of 3 months (WMD −0.19, 95% CI, −0.68 to 0.30, heterogeneity I^2^ = 0.0%), 6 months (WMD 0.06, 95% CI, −0.48 to 0.60, heterogeneity I^2^ = 77.3%) and 12 months (WMD −0.09, 95% CI, −0.65 to 0.47, heterogeneity I^2^ = 82.6%) all showed no significant difference between HoLEP and TURP based on a random effects model (Fig. S3).

#### PVR

**Figure 4:**
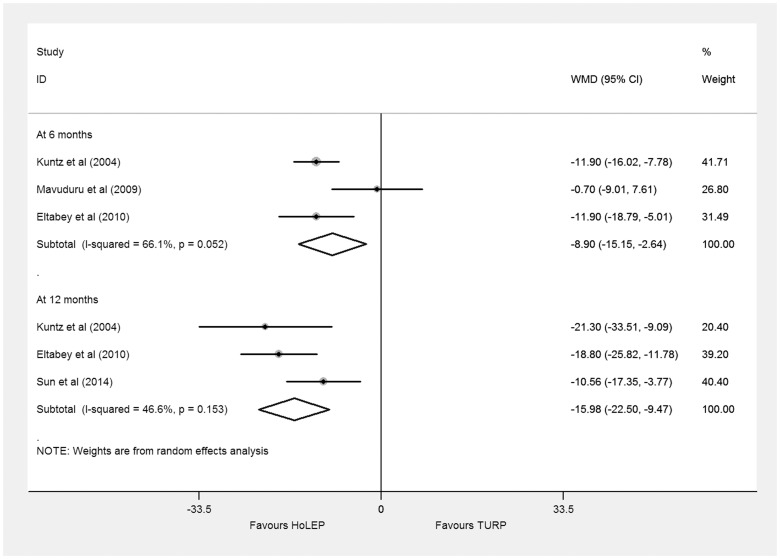
Forest plot for postvoid residual volume (PVR) at 6 months and 12 months based on a random effects model. WMD = weight mean difference; CI = confidence interval.

The PVR data were obtained from four trials including 514 BPH patients. Three trials [20], [23]–[24] reporting PVR at 6 months and three [19]–[20], [23] at 12 months were pooled with random effects model. The results presented significant differences favoring HoLEP (6 months: WMD −8.90 ml, 95% CI, −15.15 to −2.64 ml, heterogeneity I^2^ = 66.1%; 12 months: WMD −15.98 ml, 95% CI, −22.50 to −9.47 ml, heterogeneity I^2^ = 46.6%) (Fig. 4). Trial sequential adjusted 95% CI, of 6 and 12 months were −34.43 to 16.63 ml, −42.58 to 10.61 ml, respectively. Trial sequential analysis of trials data obtained at 6 and 12 months all showed that there was insufficient evidence to show a reduction of 5.0 ml in PVR, the cumulative Z-curves surpassed the futility boundary, but they did not cross the trial sequential monitoring boundary (Fig. S4, Fig. S5).

#### IEFF

Only one trial [25] reported IEFF data at 6 months (WMD 0.10, 95% CI, −1.29 to 1.49), 12 months (WMD −0.30, 95% CI, −1.73 to 1.13), and 24 months (WMD −0.30, 95% CI, −22.68 to 22.08). They were all showed no significant difference between HoLEP and TURP.

### Safety

#### Intraoperative complications

**Figure 5:**
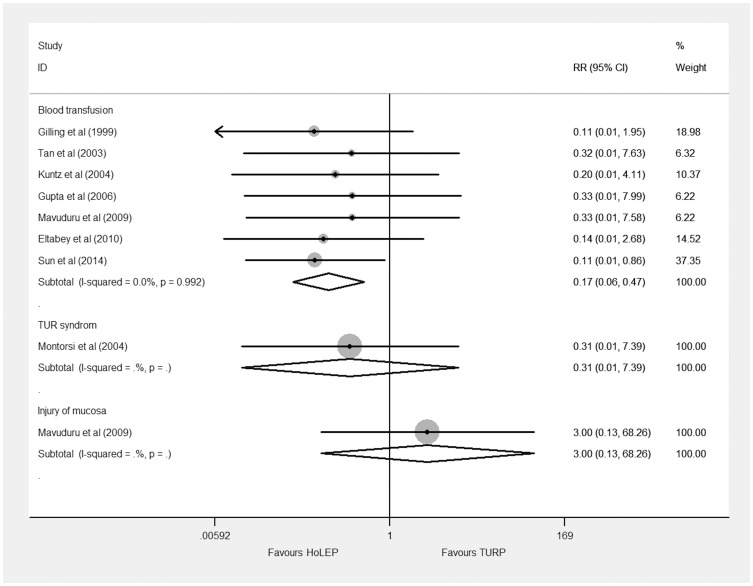
Forest plot for intraoperative complications. RR = relative risk; CI = confidence interval.

Seven trials [19]–[24], [26] reported blood transfusion involving 755 BPH patients and the result of analysis (Fig. 5) showed a significant difference between HoLEP and TURP (RR 0.17, 95% CI, 0.06 to 0.47, heterogeneity I^2^ = 0.0%). Application of a constant continuity correction of 1.0 in the zero event trial did not change the result. TSA showed that 14.8% (755) of the required information size of 5112 patients were accrued to detect or reject a 35% reduction in relative risk, the cumulative Z-curve surpassed the futility boundary, but it did not cross the trial sequential monitoring boundary (Fig. S6). The TSA adjusted 95% CI was 0.00 to 11.89.

Only one trial [25] reported TUR syndrome and one [24] reported injury of mucosa. They were both showed no significance between HoLEP and TURP (Fig. 5).

#### Early postoperative complications

**Figure 6:**
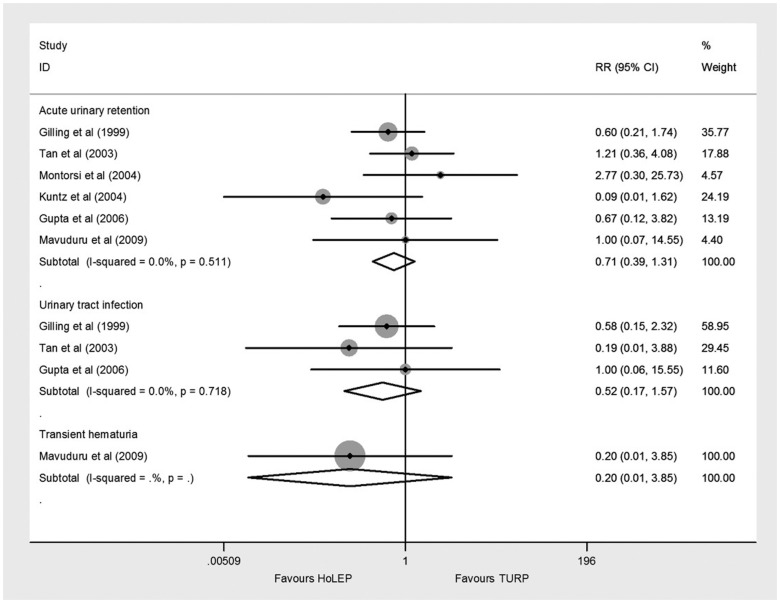
Forest plot for early postoperative complications. RR = relative risk; CI = confidence interval.

Six trials [21]–[26] reported acute urinary retention, three trials [21]–[22], [26] reported urinary tract infection, and one trial [24] reported transient hematuria. They all showed no significant difference between HoLEP and TURP (Fig. 6)

#### Late postoperative complications

**Figure 7:**
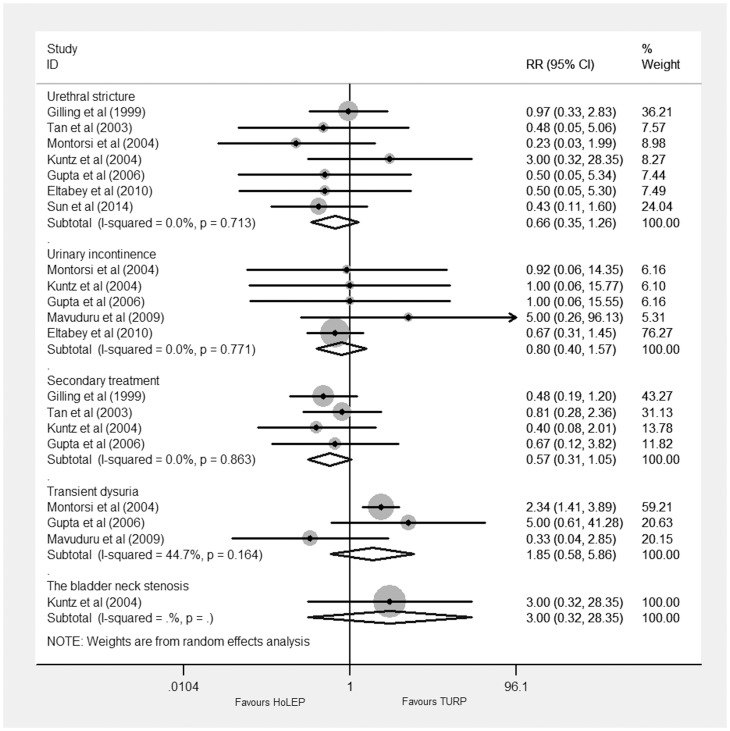
Forest plot for late postoperative complications. RR = relative risk; CI = confidence interval.

Seven trials [19]–[23], [25]–[26] reported urinary stricture, five trials [20], [22]–[25] reported urinary incontinence, four trials [21]–[23], [26] reported secondary treatment, three trials [22], [24]–[25] reported transient dysuria, and one trial [23] reported the bladder neck stenosis. They all showed no significant difference between HoLEP and TURP (Fig. 7).

### Perioperative indicators

#### Duration of operation

**Figure 8:**
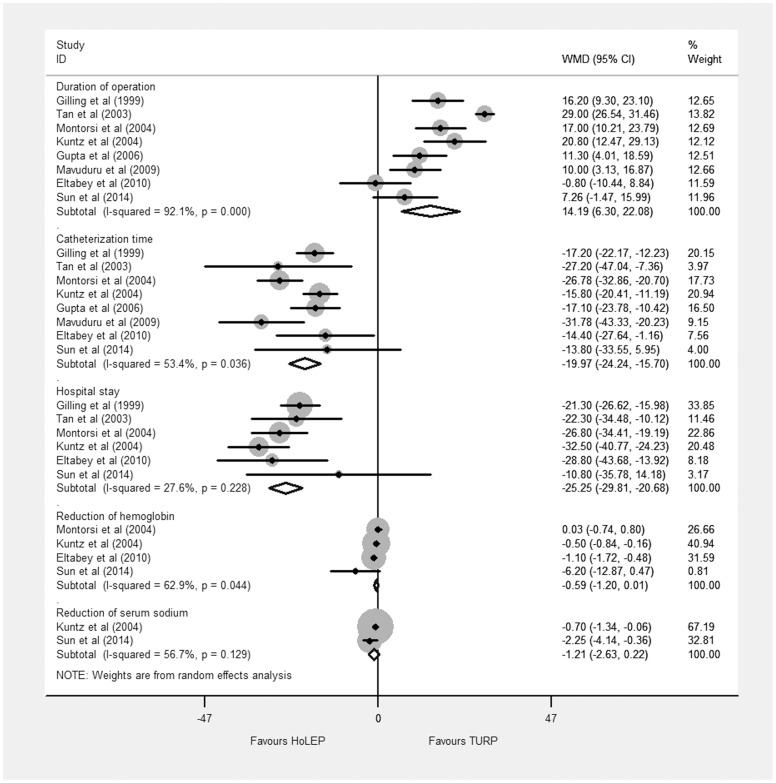
Forest plot for perioperative indicators. WMD = weight mean difference; CI = confidence interval.

**Figure 9:**
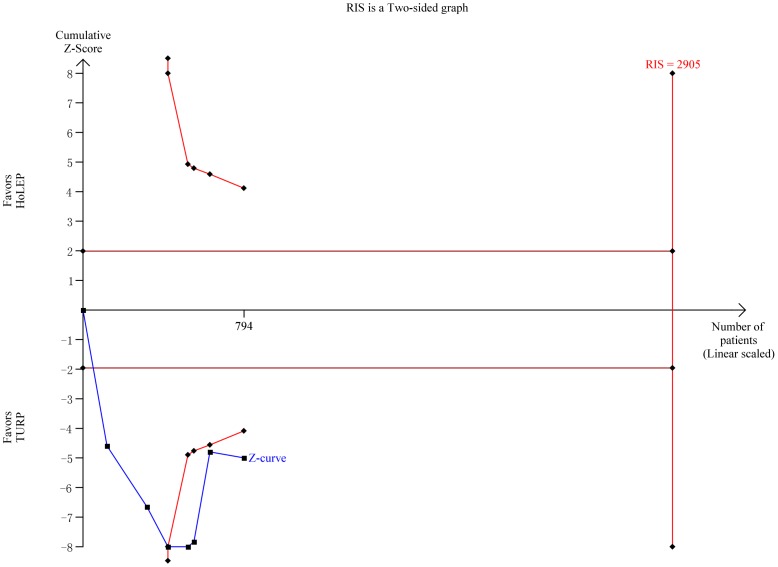
Trial sequential analysis of operation time. The required information size for operation time was calculated based on a two side α = 5%, β = 20% (power 80%), a minimal relevant difference of 5.0 min, a standard deviation of 29.2 min, and D^2^ = 63% as estimated in a random effects model.

Eight trials reported the duration of operation [19]–[26] and the pooled result showed a significant difference favoring TURP (WMD 14.19 min, 95% CI, 6.30 to 22.08 min, heterogeneity I^2^ = 92.1%; Fig. 8) based on a random effects model. TSA showed that sufficient evidence was established to show even a small reduction of 5.0 min in duration of operation, the cumulative Z-curves surpassed the futility boundary and crossed the trial sequential monitoring boundary (Fig. 9). TSA adjusted 95% CI was 2.18 to 21.99 min.

#### Catheterization time

**Figure 10:**
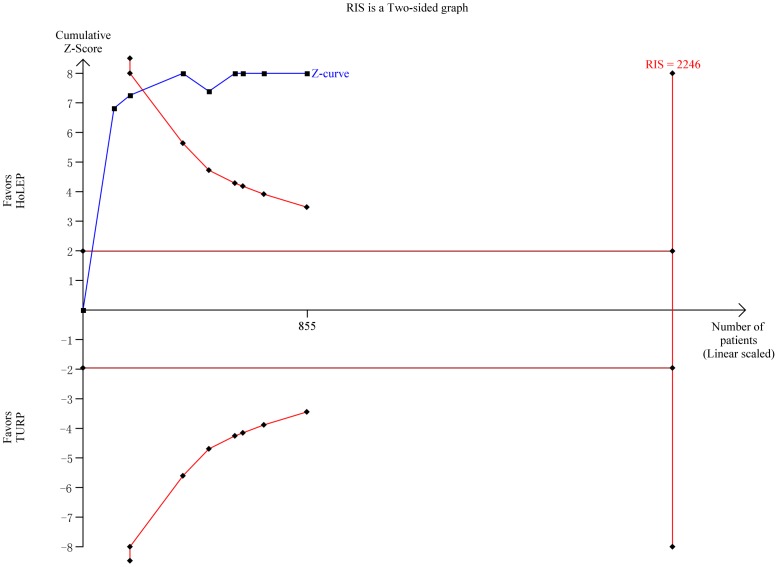
Trial sequential analysis of catheterization time. The required information size for operation time was calculated based on a two side α = 5%, β = 20% (power 80%), a minimal relevant difference of 5.0 min, a standard deviation of 26.8 min, and D^2^ = 60% as estimated in a random effects model.

The catheterization time data obtained from eight trials [19]–[26] and the meta-analysis result showed a significant difference between intervention groups (WMD −19.97 h, 95% CI, −24.24 to −15.70; heterogeneity I^2^ = 53.4%; Fig. 8) based on a random effects model. TSA showed that there was sufficient evidence to show a reduction of 5 h, with crossing of the trial sequential monitoring boundary for favoring HoLEP (Fig. 10). TSA adjusted 95% CI was −26.88 to −12.69 h.

#### Hospital stay

**Figure 11:**
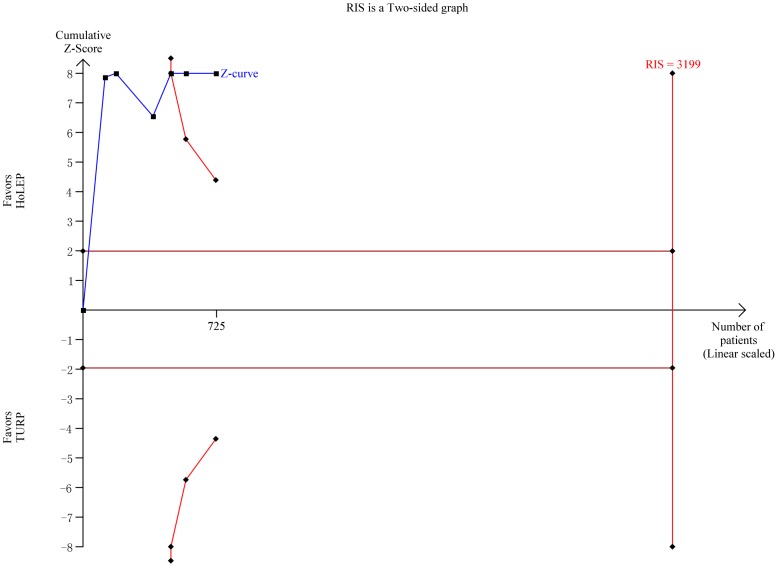
Trial sequential analysis of hospital stay. The required information size for operation time was calculated based on a two side α = 5%, β = 20% (power 80%), a minimal relevant difference of 5.0 min, a standard deviation of 34.1 min, and D^2^ = 54% as estimated in a random effects model.

Six trials [19]–[21], [23], [25]–[26] reported hospital stay data. The duration of hospital stay was shorter in HoLEP (WMD −25.25 h, 95% CI, −29.81 to −20.68 h, heterogeneity I^2^ = 27.6%; Fig. 8) based on a random effects model. TSA showed that sufficient evidence was available to show a reduction of 5 h, with crossing of the trial sequential monitoring boundary for favoring HoLEP (Fig. 11). TSA adjusted 95% CI was −35.37 to −12.13 h.

#### Reduction of hemoglobin and serum sodium

Four trials [19]–[20], [23], [25] reported reduction of hemoglobin and the pooled result showed no significant difference between HoLEP and TURP (WMD −0.59 g/dl, 95% CI, −1.20 to 0.01 g/dl; heterogeneity I^2^ = 62.9%; Fig. 8). TSA showed that there was insufficient evidence to show a reduction of 0.5 g/dl in reduction of haemoglobin, the cumulative Z-curve did not cross the trial sequential monitoring boundary (Fig. S7). TSA adjusted 95% CI was −3.07 to 1.89 g/dl.

The reporting of reduction of serum sodium was infrequent, and only two trials [19], [23] showed no significant difference between two groups (WMD −1.21 mmol/L, 95% CI, −2.63 to 0.22 mmol/L; Fig. 8).

## Discussion

### Major findings

This systematic review included a total of 8 RCTs enrolling 855 patients, all trials were assessed to be of low to moderate risk of bias. The main finding of this systematic review was that both HoLEP and TURP could significantly improve symptoms in BPH patients. There was no statistical difference between the two groups in QoL, while lower IPSS at 12 months, higher Qmax values at 3 and 12 months, less PVR at 6, 12 months were all noted in HoLEP group (P<0.05), but results of trial sequential analysis suggested evidence was not sufficient enough for the effect. Hence, we were only able to infer that HoLEP had the potential advantage in the curative effect.

In the outcome of blood transfusion, HoLEP approach was obviously better than TURP and it might be associated with better laser coagulation technology; however, trial sequential analysis did not allow us to draw any solid conclusion on safety. Only one trial reported there were no significant difference of TUR syndrome and injury of mucosa rate between HoLEP and TURP. For early and late postoperative complications, we found no significant difference in AUR/re-catheterization, UTI, transient hematuria, urethral stricture, urinary incontinence, re-operation, transient dysuria, or bladder neck stenosis. In perioperative indicators, HoLEP was associated with longer operation time. This may be due to the fact that morcellation in HoLEP requires a much longer time than traditional TURP. However, Holmium laser technique is significantly advantageous in terms of catheterization time and hospital stay. Trial sequential analysis provided firm evidence of shorter catheterization time and hospital stay associated with the treatment of HoLEP as compared to TURP.

### Results in relation to other studies and reviews

A relevant meta-analysis involving 4 RCTs by Tan et al [34] and a recent updated meta-analysis involving 6 RCTs by Yin et al [35] both reported some of the major outcomes. However, they are both associated with various weaknesses as follows: (1) incomplete study identification, which indicates lower level of efficiency in literature search and a serious risk of publication bias; (2) these published meta-analyses used Jadad Scale for assessing risk of bias, which lacks in consideration of allocation concealment and it is not recommended for use by the Cochrane Collaboration [8]; (3) small sample sizes. In addition, their studies were not registered, and the main results of effectiveness evaluation (Qmax) were different [34]–[35].

Another earlier review/meta-analysis [36] showed the most commonly minimally invasive surgical therapy (MIST) for BPH at that time. But only 4 included trials compared HoLEP with TURP, and the authors did not explore HoLEP vs. TURP in greater depth. Other shortcomings of this study included a lack of the methodological quality assessment tool for the included RCTs, and there were no subgroup analyses of effective outcomes according to follow-up time. In addition, there was no information on perioperative outcomes such as hospital stay.

### Strengths and limitations

Compared with previous meta-analyses, our systematic review has several strengths. First, we based it on a published protocol with rigid inclusion criteria for randomized clinical trials (http://www.crd.york.ac.uk/PROSPERO/display_record.asp?ID=CRD42014007334). Second, our study included 8 RCTs and considered more outcomes, which can provide a more comprehensive view on the efficacy and safety. Third, our study followed the recommended Cochrane collaboration's tool for assessing risk of bias. The previous meta-analyses [34]–[35] used the Jadad Scale, which lacks in consideration of allocation concealment and is not recommended by the Cochrane Handbook for Systematic Reviews of Interventions [8]. Therefore, results of the methodological quality assessment of our study are more robust. Fourth, our search strategy was devised rigorously with a more precise focus and we placed no restrictions on the type of outcomes reported in the trials (Appendix S1); therefore, we found more eligible RCTs. Fifth, we attempted to evaluate the strength of the available evidence with comprehensive analyses of the risk of bias using subgroup analyses with test for subgroup differences and also applied the new method that called “trial sequential analysis” to identify whether the outcomes reach a conclusive conclusion [10]–[11], [15], [37]. To our knowledge, this is the first application trial sequential analysis in Urology. And we added results of sexual function.

Our study has some limitations that should be demonstrated. We contacted corresponding authors of all trials to clarify methodological details and obtain relevant outcomes, but only a few authors responded. Therefore, firstly, the precise methodological quality of the included studies remains unclear. Secondly, since most of the included RCTs lacked long-term data (>12 months), we were unable to provide any long-term evidence. Thirdly, data were sparse for sexual function. Fourthly, the included studies do not provide enough information as to prostate size and anti-coagulated patients for in-depth subgroup analysis. Lastly, the overall sample size was still small.

### Implication for research and practice

Our meta-analysis may also have some implications for further researches and clinical practice. Future researches should clarify the effectiveness, safety, potential advantages and disadvantages of HoLEP compared with TURP in large, high-quality RCTs, which also evaluate long-term outcomes and sexual functions relevant outcomes and focus more on prostate size, anti-coagulated patients and so on. In clinical practice, surgeons should not be limited to only conventional TURP as a treatment option for BPH. Although conventional TURP is still regarded as “gold-standard” in clinical guidelines, our findings have illustrated several advantages of HoLEP including a more favorable procedural safety profile, shorter catheterization duration time and hospital stay. We would thus like to highlight to clinicians that HoLEP presents as a viable treatment option for BPH. It is potentially a better treatment strategy, especially for elderly patients, those with large volume of prostate or high risk patients.

## Conclusions

In summary, our study provided the strongest available evidence and showed that there were no clinically relevant differences in early and late postoperative complications between the two techniques. Although the operative time favored TURP, HoLEP was more preferable due to its more favorable profile, defined by the clinically relevant differences detected regarding curative effect and less blood transfusion. Additionally, catheterization time and hospital stay were significantly shorter in HoLEP. After TSA adjustment for sparse data and multiple updating in cumulative meta-analysis, it seems unsure that HoLEP provides overall clinical benefit for BPH patients. Considering our main limitations, data from large, well-conducted international/multicentre RCTs with long-term data (follow-up duration>12 months) are necessary; sexual function-analysis and cost-analysis are still needed, and the comparison between the two approaches remains open.

## Supporting Information

Figure S1 **Trial sequential analysis of International Prostate Symptom Score (IPSS) at 12 months.** The required information size for IPSS at 12 months was calculated based on a two side α = 5%, β = 20% (power 80%), a minimal relevant difference of 0.5, a standard deviation of 3.5, and D^2^ = 77% as estimated in a random effects model.
(TIF)

Figure S2 **Trial sequential analysis of maximum flow rate (Qmax) at 3 months.** The required information size for Qmax at 3 months was calculated based on a two side α = 5%, β = 20% (power 80%), a minimal relevant difference of 3.0 ml/s, a standard deviation of 13.8 ml/s, and D^2^ = 0% as estimated in a fixed effects model.
(TIF)

Figure S3 **Forest plot for quality of life (QoL) at 3 months, 6 months, and 12 months based on a random effects model.** WMD = weight mean difference; CI = confidence interval.
(TIF)

Figure S4 **Trial sequential analysis of postvoid residual volume (PVR) at 6 months.** The required information size for PVR at 6 months was calculated based on a two side α = 5%, β = 20% (power 80%), a minimal relevant difference of 5.0 ml, a standard deviation of 20.7 ml, and D^2^ = 73% as estimated in a random effects model.
(TIF)

Figure S5 **Trial sequential analysis of postvoid residual volume (PVR) at 12 months.** The required information size for PVR at 6 months was calculated based on a two side α = 5%, β = 20% (power 80%), a minimal relevant difference of 5.0 ml, a standard deviation of 36.7 ml, and D^2^ = 0% as estimated in a random effects model.
(TIF)

Figure S6 **Trial sequential analysis of blood transfusion.** A diversity adjusted information size of 5112 patients was calculated using a two side α = 5%, β = 20% (power 80%), D^2^ = 0%, an anticipated relative risk increase of 35% and an event proportion of 4% in the control arm. Trials with no events were included in the study with a constant continuity correction of 1. The blue cumulative Z-curve was constructed using a fixed effects model.
(TIF)

Figure S7 **Trial sequential analysis of hemoglobin decrease.** The required information size for operation time was calculated based on a two side α = 5%, β = 20% (power 80%), a minimal relevant difference of 0.5 g/dl, a standard deviation of 2.3 g/dl, and D^2^ = 79% as estimated in a random effects model.
(TIF)

Appendix S1 **Search strategy protocols used for each electronic database.**
(DOC)

Checklist S1 **PRISMA checklist.**
(DOC)

## References

[1] McVary KT, Roehrborn CG, Avins AL, Barry MJ, Bruskewitz RC, et al (2011) Update on AUA guideline on the management of benign prostatic hyperplasia. J Urol 185: 1793–1803. 21420124 10.1016/j.juro.2011.01.074

[2] Oelke M, Bachmann A, Descazeaud A, Emberton M, Gravas S, et al (2013) EAU guidelines on the treatment and follow-up of non-neurogenic male lower urinary tract symptoms including benign prostatic obstruction. Eur Urol 64: 118–140. 23541338 10.1016/j.eururo.2013.03.004

[3] Gilling P, Fraundorfer M, Kabalin J (1997) Holmium: YAG laser resection of the prostate (HoLRP) versus transurethral electrocautery resection of the prostate (TURP): a prospective randomized, urodynamics-based clinical trial. J Urol 157: 149A.

[4] Gilling PJ, Aho TF, Frampton CM, King CJ, Fraundorfer MR (2008) Holmium laser enucleation of the prostate: results at 6 years. Eur Urol 53: 744–749. 17475395 10.1016/j.eururo.2007.04.052

[5] Kabalin JN, Mackey MJ, Cresswell MD, Fraundorfer MR, Gilling PJ (1997) Holmium: YAG laser resection of prostate (HoLRP) for patients in urinary retention. J Endourol 11: 291–293. 9376851 10.1089/end.1997.11.291

[6] Kim SH, Yoo C, Choo M, Paick JS, Oh SJ (2014) Factors affecting de novo urinary retention after Holmium laser enucleation of the prostate. PLoS One 9: e84938. 24465454 10.1371/journal.pone.0084938 PMC3897383

[7] Moher D, Liberati A, Tetzlaff J, Altman DG (2009) Group P (2009) Preferred reporting items for systematic reviews and meta-analyses: the PRISMA statement. PLoS Med 6: e1000097. 19621072 10.1371/journal.pmed.1000097 PMC2707599

[8] Higgins JP, Altman DG, Gotzsche PC, Juni P, Moher D, et al (2011) The Cochrane Collaboration's tool for assessing risk of bias in randomised trials. BMJ 343: d5928. 22008217 10.1136/bmj.d5928 PMC3196245

[9] JPT CHH, Green S (2011) Cochrane handbook for systematic reviews of interventions version 5.1. 0 [updated March 2011]. The Cochrane Collaboration

[10] Brok J, Thorlund K, Gluud C, Wetterslev J (2008) Trial sequential analysis reveals insufficient information size and potentially false positive results in many meta-analyses. J Clin Epidemiol 61: 763–769. 18411040 10.1016/j.jclinepi.2007.10.007

[11] Brok J, Thorlund K, Wetterslev J, Gluud C (2009) Apparently conclusive meta-analyses may be inconclusive–Trial sequential analysis adjustment of random error risk due to repetitive testing of accumulating data in apparently conclusive neonatal meta-analyses. Int J Epidemiol 38: 287–298. 18824466 10.1093/ije/dyn188

[12] Thorlund K, Devereaux PJ, Wetterslev J, Guyatt G, Ioannidis JP, et al (2009) Can trial sequential monitoring boundaries reduce spurious inferences from meta-analyses? Int J Epidemiol 38: 276–286. 18824467 10.1093/ije/dyn179

[13] Thorlund K, Engstrøm J, Wetterslev J, Brok J, Imberger G, et al. (2011) User manual for trial sequential analysis (TSA). Copenhagen Trial Unit Available: http://wwwctudk/tsa/files/tsa_manualpdf. Accessed 2014 Feb 26.

[14] Thorlund K, Imberger G, Walsh M, Chu R, Gluud C, et al (2011) The number of patients and events required to limit the risk of overestimation of intervention effects in meta-analysis–a simulation study. PLoS One 6: e25491. 22028777 10.1371/journal.pone.0025491 PMC3196500

[15] Wetterslev J, Thorlund K, Brok J, Gluud C (2008) Trial sequential analysis may establish when firm evidence is reached in cumulative meta-analysis. J Clin Epidemiol 61: 64–75. 18083463 10.1016/j.jclinepi.2007.03.013

[16] Wetterslev J, Thorlund K, Brok J, Gluud C (2009) Estimating required information size by quantifying diversity in random-effects model meta-analyses. BMC Med Res Methodol 9: 86. 20042080 10.1186/1471-2288-9-86 PMC2809074

[17] Lan KG, DeMets DL (1983) Discrete sequential boundaries for clinical trials. Biometrika 70: 659–663.

[18] Higgins JP, Whitehead A, Simmonds M (2011) Sequential methods for random-effects meta-analysis. Stat Med 30: 903–921. 21472757 10.1002/sim.4088 PMC3107948

[19] Sun N, Fu Y, Tian T, Gao J, Wang Y, et al (2014) Holmium laser enucleation of the prostate versus transurethral resection of the prostate: a randomized clinical trial. Int Urol Nephrol 10.1007/s11255-014-0646-9 24492988

[20] Eltabey MA, Sherif H, Hussein AA (2010) Holmium laser enucleation versus transurethral resection of the prostate. Can J Urol 17: 5447–5452. 21172109

[21] Gilling PJ, Mackey M, Cresswell M, Kennett K, Kabalin JN, et al (1999) Holmium laser versus transurethral resection of the prostate: a randomized prospective trial with 1-year followup. The Journal of urology 162: 1640–1644. 10524887

[22] Gupta N, Sivaramakrishna, Kumar R, Dogra PN, Seth A (2006) Comparison of standard transurethral resection, transurethral vapour resection and holmium laser enucleation of the prostate for managing benign prostatic hyperplasia of >40 g. BJU international 97: 85–89. 16336334 10.1111/j.1464-410X.2006.05862.x

[23] Kuntz RM, Ahyai S, Lehrich K, Fayad A (2004) Transurethral holmium laser enucleation of the prostate versus transurethral electrocautery resection of the prostate: a randomized prospective trial in 200 patients. J Urol 172: 1012–1016. 15311026 10.1097/01.ju.0000136218.11998.9e

[24] Mavuduru RM, Mandal AK, Singh SK, Acharya N, Agarwal M, et al (2009) Comparison of HoLEP and TURP in terms of efficacy in the early postoperative period and perioperative morbidity. Urol Int 82: 130–135. 19321996 10.1159/000200786

[25] Montorsi F, Naspro R, Salonia A, Suardi N, Briganti A, et al (2004) Holmium laser enucleation versus transurethral resection of the prostate: results from a 2-center, prospective, randomized trial in patients with obstructive benign prostatic hyperplasia. J Urol 172: 1926–1929. 15540757 10.1097/01.ju.0000140501.68841.a1

[26] Tan AH, Gilling PJ, Kennett KM, Frampton C, Westenberg AM, et al (2003) A randomized trial comparing holmium laser enucleation of the prostate with transurethral resection of the prostate for the treatment of bladder outlet obstruction secondary to benign prostatic hyperplasia in large glands (40 to 200 grams). J Urol 170: 1270–1274. 14501739 10.1097/01.ju.0000086948.55973.00

[27] Ahyai SA, Lehrich K, Kuntz RM (2007) Holmium laser enucleation versus transurethral resection of the prostate: 3-year follow-up results of a randomized clinical trial. Eur Urol 52: 1456–1463. 17499427 10.1016/j.eururo.2007.04.053

[28] Fraundorfer MR, Gilling PJ, Kennett KM, Dunton NG (2001) Holmium laser resection of the prostate is more cost effective than transurethral resection of the prostate: results of a randomized prospective study. Urology 57: 454–458. 11248619 10.1016/s0090-4295(00)00987-0

[29] Gilling PJ, Wilson LC, King CJ, Westenberg AM, Frampton CM, et al (2012) Long-term results of a randomized trial comparing holmium laser enucleation of the prostate and transurethral resection of the prostate: results at 7 years. BJU Int 109: 408–411. 21883820 10.1111/j.1464-410X.2011.10359.x

[30] Rigatti L, Naspro R, Salonia A, Centemero A, Ghezzi M, et al (2006) Urodynamics after TURP and HoLEP in urodynamically obstructed patients: Are there any differences at 1 year of follow-up? Urology 67: 1193–1198. 16750253 10.1016/j.urology.2005.12.036

[31] Westenberg A, Gilling P, Kennett K, Frampton C, Fraundorfer M (2004) Holmium laser resection of the prostate versus transurethral resection of the prostate: results of a randomized trial with 4-year minimum long-term followup. J Urol 172: 616–619. 15247745 10.1097/01.ju.0000132739.57555.d8

[32] Wilson LC, Gilling PJ, Williams A, Kennett KM, Frampton CM, et al (2006) A randomised trial comparing holmium laser enucleation versus transurethral resection in the treatment of prostates larger than 40 grams: results at 2 years. Eur Urol 50: 569–573. 16704894 10.1016/j.eururo.2006.04.002

[33] Briganti A, Naspro R, Gallina A, Salonia A, Vavassori I, et al (2006) Impact on sexual function of holmium laser enucleation versus transurethral resection of the prostate: results of a prospective, 2-center, randomized trial. J Urol 175: 1817–1821. 16600770 10.1016/S0022-5347(05)00983-3

[34] Tan A, Liao C, Mo Z, Cao Y (2007) Meta-analysis of holmium laser enucleation versus transurethral resection of the prostate for symptomatic prostatic obstruction. Br J Surg 94: 1201–1208. 17729384 10.1002/bjs.5916

[35] Yin L, Teng J, Huang CJ, Zhang X, Xu D (2013) Holmium laser enucleation of the prostate versus transurethral resection of the prostate: a systematic review and meta-analysis of randomized controlled trials. J Endourol 27: 604–611. 23167266 10.1089/end.2012.0505

[36] Ahyai SA, Gilling P, Kaplan SA, Kuntz RM, Madersbacher S, et al (2010) Meta-analysis of functional outcomes and complications following transurethral procedures for lower urinary tract symptoms resulting from benign prostatic enlargement. European Urology 58: 384–397. 20825758 10.1016/j.eururo.2010.06.005

[37] Hemmingsen B, Christensen LL, Wetterslev J, Vaag A, Gluud C, et al (2012) Comparison of metformin and insulin versus insulin alone for type 2 diabetes: systematic review of randomised clinical trials with meta-analyses and trial sequential analyses. BMJ 344: e1771. 22517929 10.1136/bmj.e1771

