# Supplementary figures and images for: Holmium Laser Enucleation versus Transurethral Resection in Patients with Benign Prostate Hyperplasia: An Updated Systematic Review with Meta-Analysis and Trial Sequential Analysis
Source: PLoS One. 2014 Jul 8;9(7):e101615. doi: 10.1371/journal.pone.0101615 (PMC4086899; doi:10.1371/journal.pone.0101615)

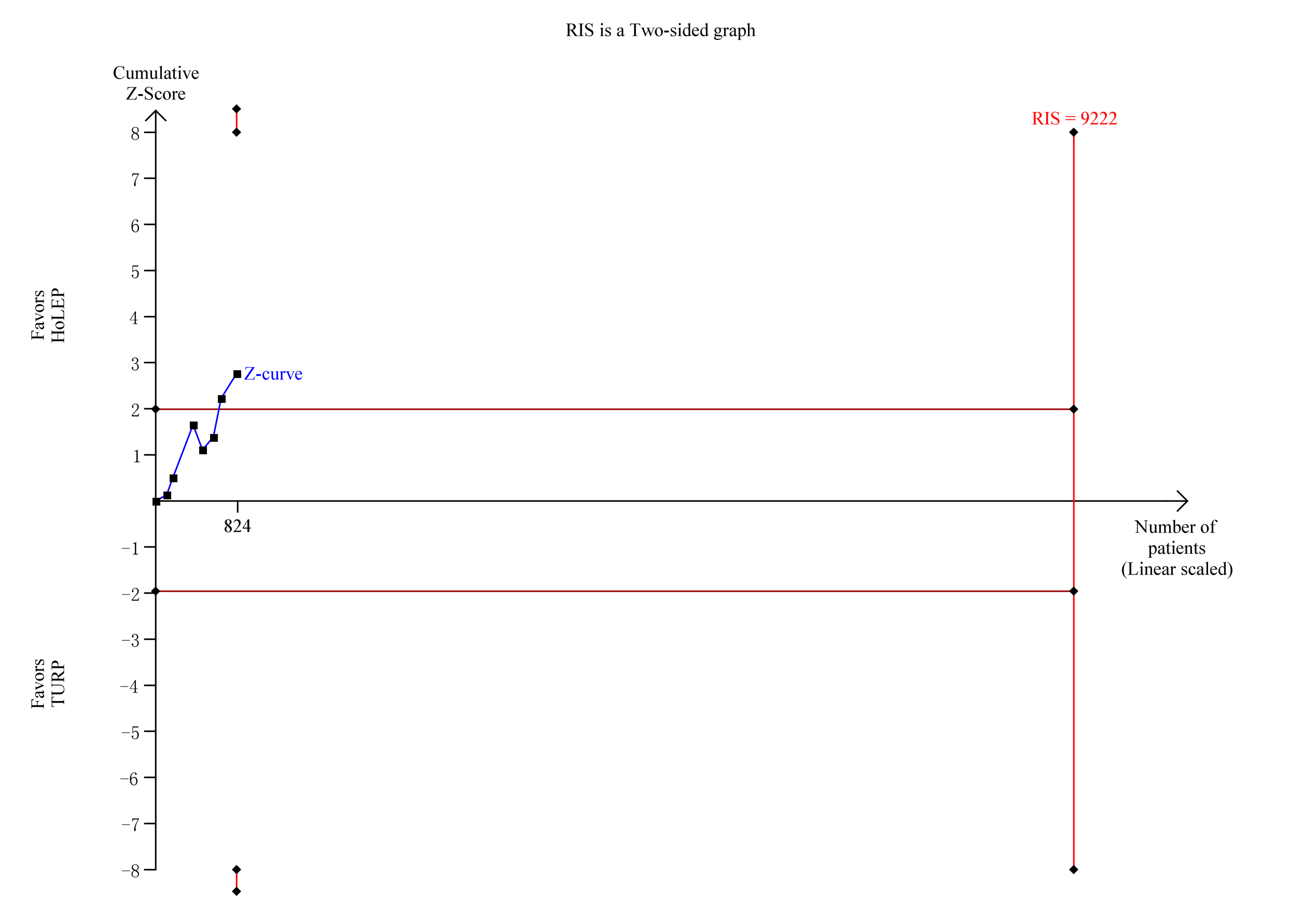

Supplement: Figure S1 — Trial sequential analysis of International Prostate Symptom Score (IPSS) at 12 months. The required information size for IPSS at 12 months was calculated based on a two side α = 5%, β = 20% (power 80%), a minimal relevant difference of 0.5, a standard deviation of 3.5, and D2 = 77% as estimated in a random effects model. (TIF) [file pone.0101615.s001.tif]

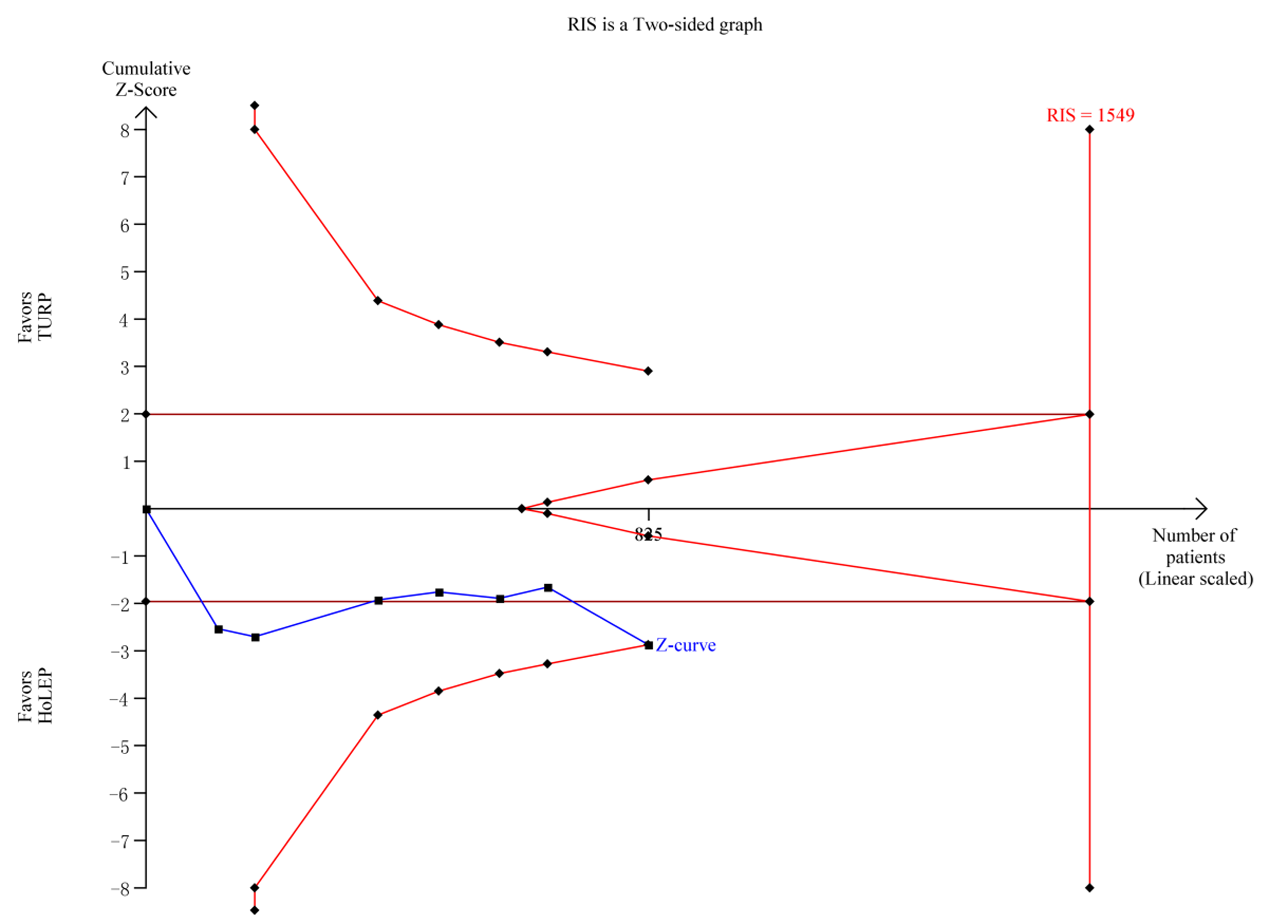

Supplement: Figure S2 — Trial sequential analysis of maximum flow rate (Qmax) at 3 months. The required information size for Qmax at 3 months was calculated based on a two side α = 5%, β = 20% (power 80%), a minimal relevant difference of 3.0 ml/s, a standard deviation of 13.8 ml/s, and D2 = 0% as estimated in a fixed effects model. (TIF) [file pone.0101615.s002.tif]

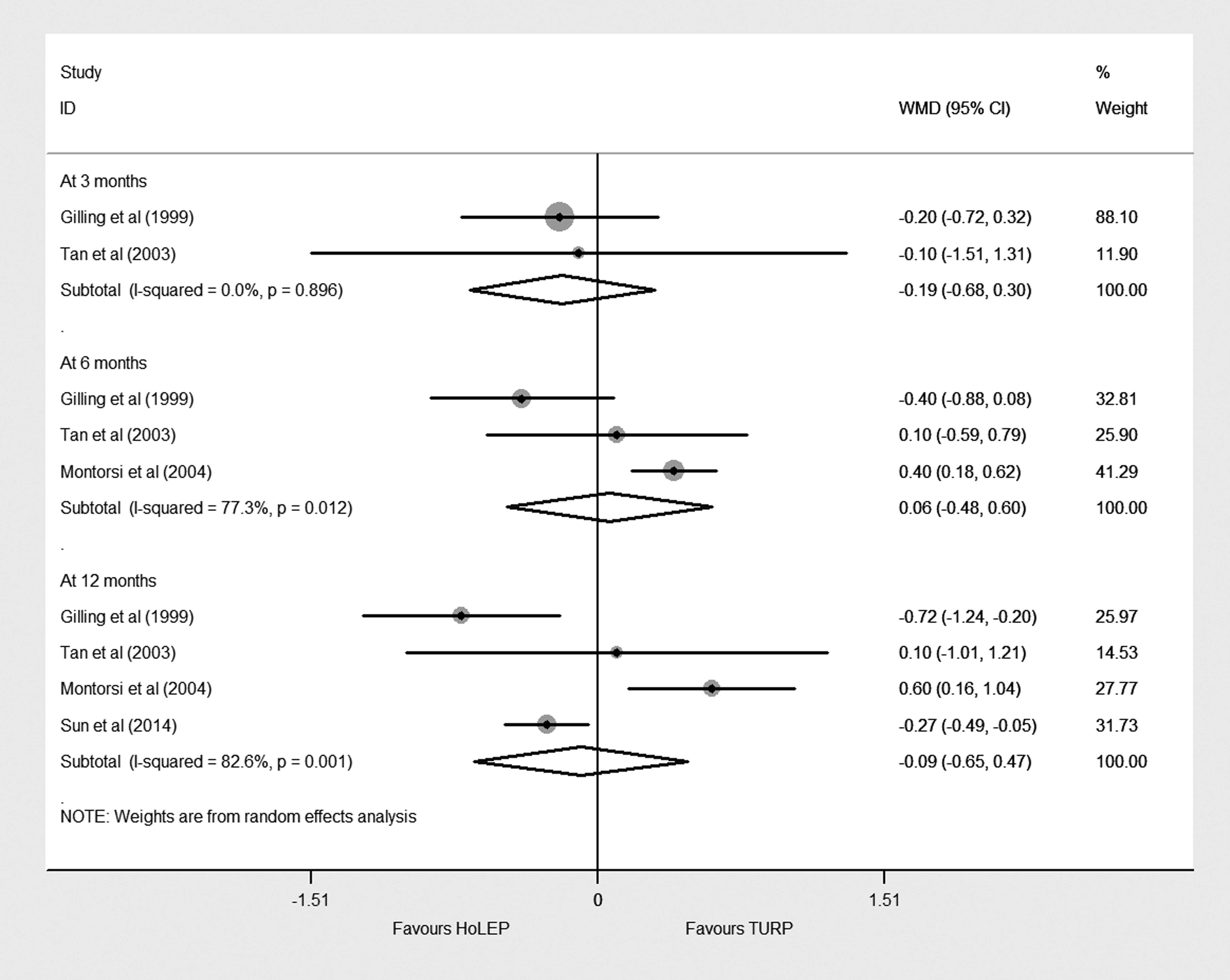

Supplement: Figure S3 — Forest plot for quality of life (QoL) at 3 months, 6 months, and 12 months based on a random effects model. WMD = weight mean difference; CI = confidence interval. (TIF) [file pone.0101615.s003.tif]

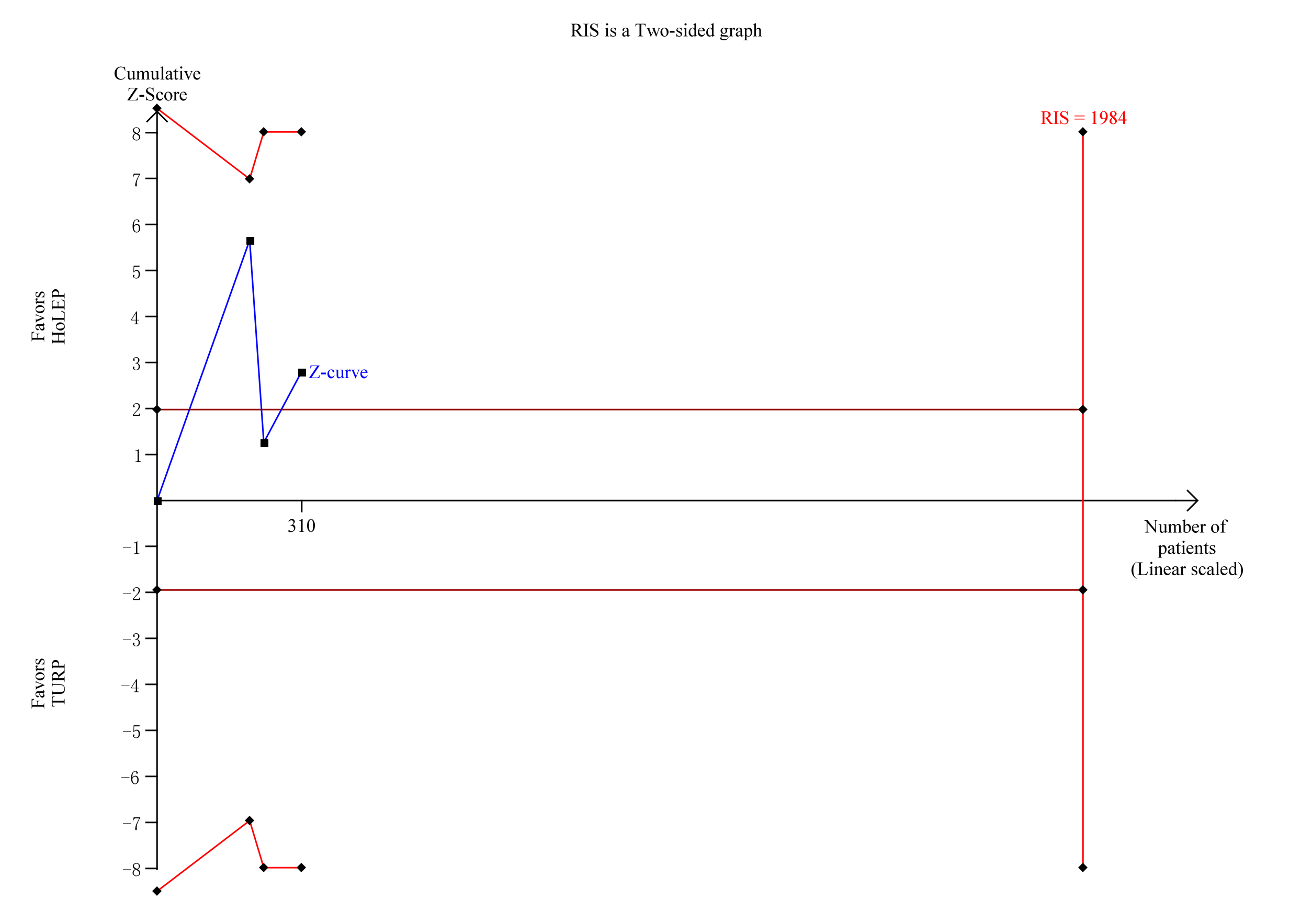

Supplement: Figure S4 — Trial sequential analysis of postvoid residual volume (PVR) at 6 months. The required information size for PVR at 6 months was calculated based on a two side α = 5%, β = 20% (power 80%), a minimal relevant difference of 5.0 ml, a standard deviation of 20.7 ml, and D2 = 73% as estimated in a random effects model. (TIF) [file pone.0101615.s004.tif]

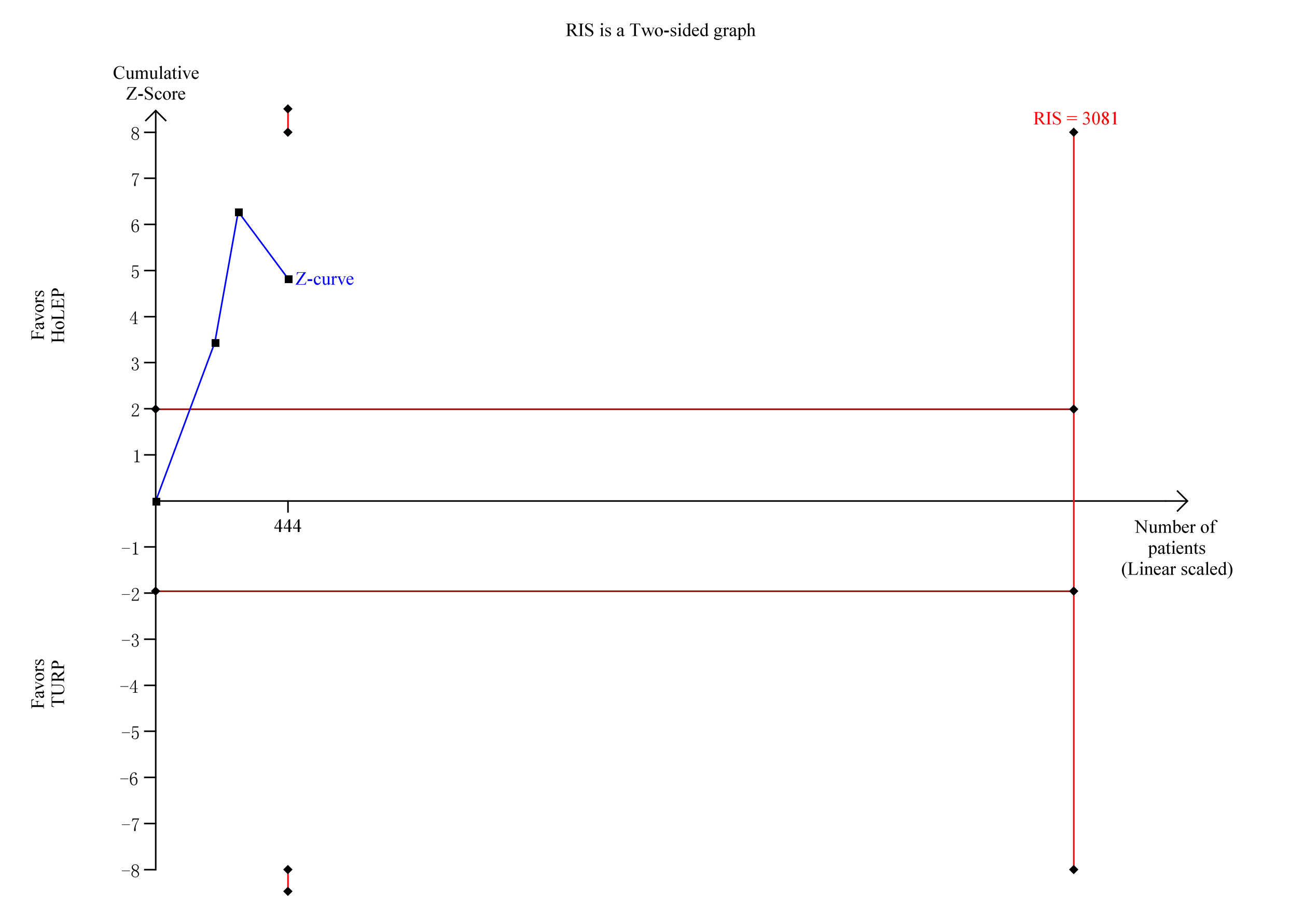

Supplement: Figure S5 — Trial sequential analysis of postvoid residual volume (PVR) at 12 months. The required information size for PVR at 6 months was calculated based on a two side α = 5%, β = 20% (power 80%), a minimal relevant difference of 5.0 ml, a standard deviation of 36.7 ml, and D2 = 0% as estimated in a random effects model. (TIF) [file pone.0101615.s005.tif]

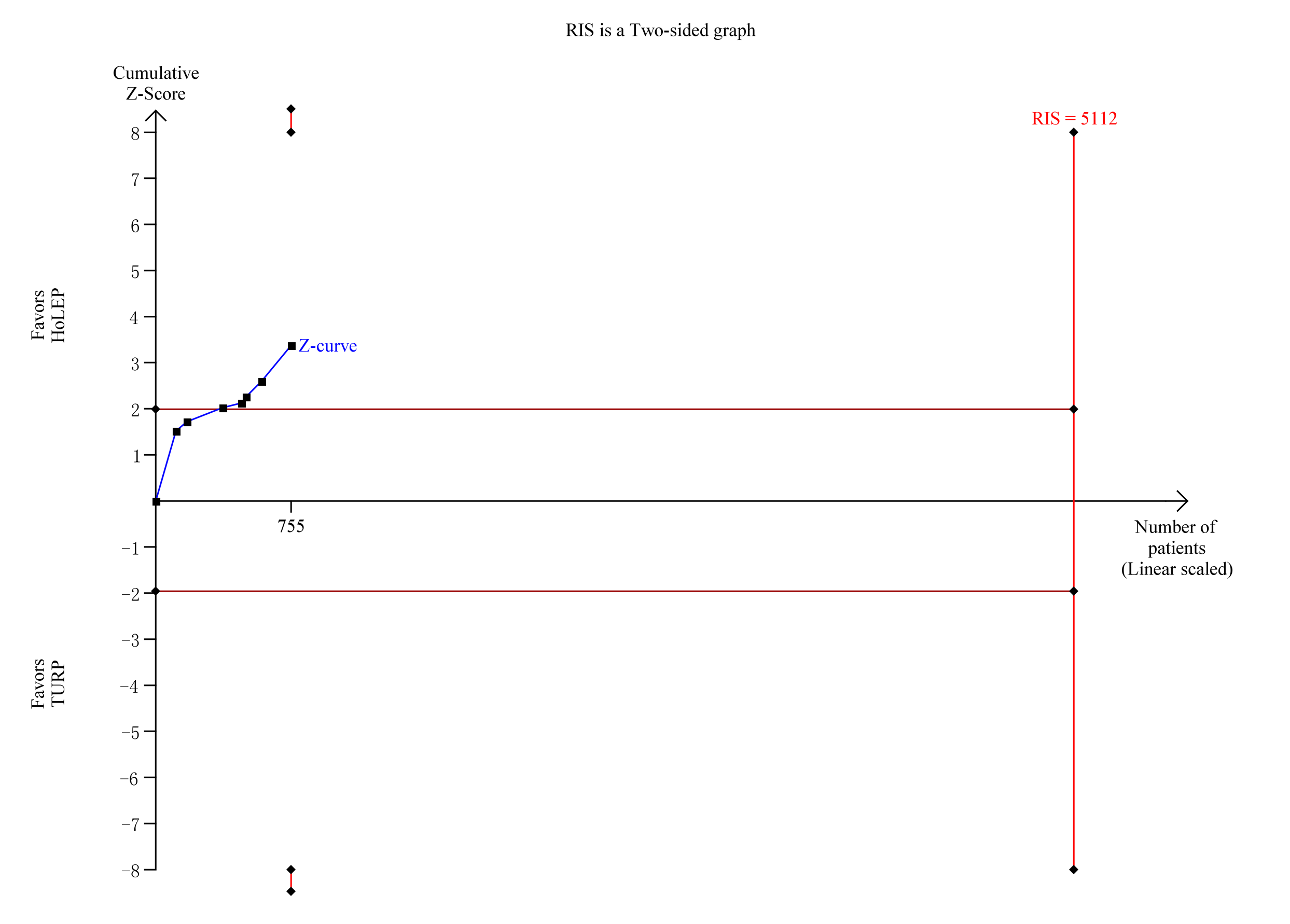

Supplement: Figure S6 — Trial sequential analysis of blood transfusion. A diversity adjusted information size of 5112 patients was calculated using a two side α = 5%, β = 20% (power 80%), D2 = 0%, an anticipated relative risk increase of 35% and an event proportion of 4% in the control arm. Trials with no events were included in the study with a constant continuity correction of 1. The blue cumulative Z-curve was constructed using a fixed effects model. (TIF) [file pone.0101615.s006.tif]

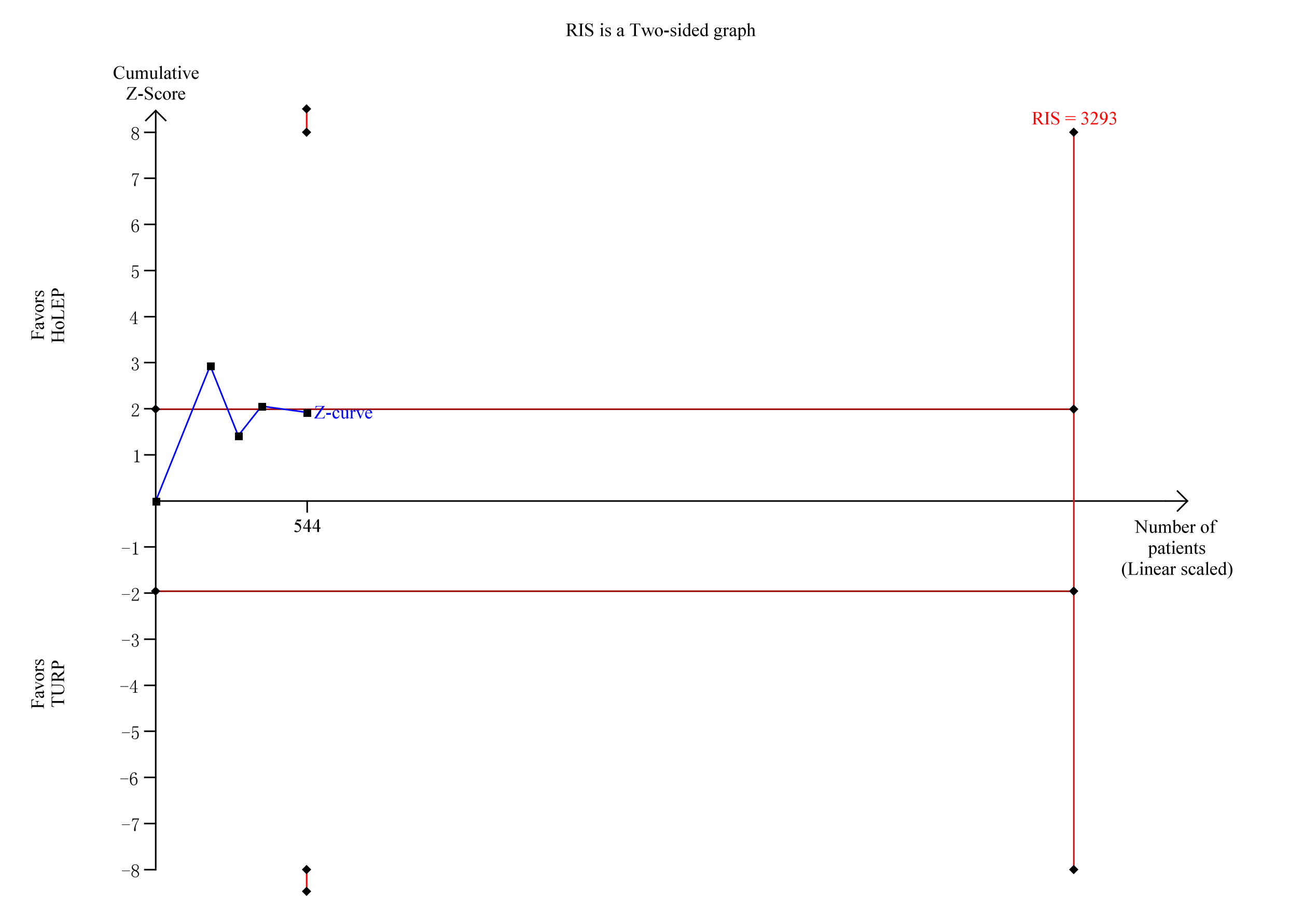

Supplement: Figure S7 — Trial sequential analysis of hemoglobin decrease. The required information size for operation time was calculated based on a two side α = 5%, β = 20% (power 80%), a minimal relevant difference of 0.5 g/dl, a standard deviation of 2.3 g/dl, and D2 = 79% as estimated in a random effects model. (TIF) [file pone.0101615.s007.tif]
