# Supplementary material for: Holmium Laser Enucleation versus Transurethral Resection in Patients with Benign Prostate Hyperplasia: An Updated Systematic Review with Meta-Analysis and Trial Sequential Analysis
Source: PLoS One. 2014 Jul 8;9(7):e101615. doi: 10.1371/journal.pone.0101615 (PMC4086899; doi:10.1371/journal.pone.0101615)
Supplement: Appendix S1 — Search strategy protocols used for each electronic database. (DOC) [file pone.0101615.s008.doc]

**Appendix A. Search strategy protocols used for each electronic database**

(1) PubMed:

(ablative[All Fields] OR "minimally invasive"[All Fields] OR "Holmium*"[All Fields] OR "Ho:YAG"[All Fields] OR "HoLEP"[All Fields] OR "HoLRP"[All Fields] OR "HoLAP"[All Fields]) AND ("TURP"[All Fields] OR "transurethral prostatectom*"[All Fields] OR "transurethral prostate resection*"[All Fields] OR "Transurethral Resection of Prostate"[Mesh] OR "transurethral resection*"[All Fields]) AND (((((((((((((((((((volunteer*[tw]) OR ((prospectiv*[tw]))) OR ((control*[tw]))) OR ((prospective studies[mh]))) OR ((follow-up studies[mh]))) OR ((evaluation studies[pt]))) OR ((comparative study[pt]))) OR ((research design[mh:noexp]))) OR ((random*[tw]))) OR (((singl* OR doubl* OR trebl* OR tripl*) AND (blind* OR mask*)))) OR ((clinical trial[tw]))) OR ((clinical trials[mh]))) OR ((clinical trial[pt]))) OR ((single-blind method[mh]))) OR ((double blind method[mh]))) OR ((random allocation[mh]))) OR ((randomized clinical trials[mh]))) OR ((controlled clinical trial[pt]))) OR ((randomized controlled trial[pt])))

(2)The Cochrane Library:

('ablative' OR 'minimally invasive' OR Holmium* OR 'HoLEP' OR 'HoLRP' OR 'HoLAP'):ti,ab,kw AND ('TURP' OR transurethral prostatectom* OR transurethral prostate resection* OR transurethral resection*) :ti,ab,kw

(3) Science Citation Index:

((TS=(((((ablative OR minimally invasive) OR Holmium*) OR HoLEP) OR HoLRP) OR HoLAP) AND TS=(((TURP OR Transurethral prostatectom*) OR Transurethral prostate resection) OR transurethral resection*)) AND TS=((Trial OR trials) OR random*))

(4) EMBASE:

(ablative OR 'minimally invasive' OR Holmium* OR HoLEP OR HoLRP OR HoLAP) AND ('transurethral resection'/exp OR 'transurethral resection of the prostate' OR 'transurethral prostate resection' OR TURP OR transurethral prostatectom$) AND ('Clinical Trial'/exp OR 'controlled clinical trial'/exp OR random$ OR trial*)
